# Evaluation of maize lodging resistance based on the critical wind speed of stalk breaking during the late growth stage

**DOI:** 10.1186/s13007-020-00689-z

**Published:** 2020-11-04

**Authors:** Jun Xue, Bo Ming, Ruizhi Xie, Keru Wang, Peng Hou, Shaokun Li

**Affiliations:** 1 Institute of Crop Sciences, Chinese Academy of Agricultural Sciences, Beijing, 100081 China; 2 Key Laboratory of Crop Physiology and Ecology, Ministry of Agriculture and Rural Affairs, Beijing, 100081 China

**Keywords:** Maize, Stalk lodging, Critical wind speed, Stalk strength, Cultivar

## Abstract

**Background:**

The accurate evaluation of the stalk-lodging resistance during the late stage of maize growth can provide a basis for the selection of cultivars, the evaluation of cultivation techniques, and timely mechanical grain harvesting. In this study, the critical wind speed of stalk breaking, plant morphology, stalk mechanical strength, and lodging rate were investigated in 10 maize cultivars to identify the parameters evaluate lodging resistance during the later growth stage of maize. Clarify the relationship with the stalk mechanical strength, critical wind speed of stalk breaking, and natural lodging rate in the field.

**Results:**

The results showed that, in the late growth stage, with increasing number of days after physiological maturity, (1) the stalk lodging rate gradually increased, (2) the stalk breaking force and rind penetration strength (RPS) of the third internode above the soil gradually decreased, and (3) the critical wind speed of stalk breaking increased first and then decreased, and was highest at about 16–24 days after physiological maturity. The position of stalk lodging mostly occurred between second and fifth internodes. The torque at the base of maize plant increased as wind speed increased, and the different of torque was excited among different maize cultivars under same wind speed. Furthermore, the stalk lodging rate was significantly negatively correlated with the critical wind speed of stalk breaking. Additionally, the critical wind speed of stalk breaking was significantly positively correlated with the stalk breaking force and the RPS.

**Conclusion:**

This indicates that the critical wind speed of stalk breaking is a superior way to determine the stalk lodging resistance. These results suggest that, in the late growth stage, the decrease in the stalk mechanical strength is an important reason for the decrease in the critical wind speed of stalk breaking and the increase in the lodging rate.

## Background

Crop lodging can lead to the physical collapse of the plant canopy and can happen spontaneously due to mechanical instability of the plant structure, through external forces such as wind, or both. Maize lodging can occur at both the stalk and root. Stalk lodging occurs when stalks are broken at or below the ear-bearing node, whereas root lodging refers to plants that lean at an angle greater than a certain threshold (typically 30 or 45°) from the vertical [1, 2]. Maize plants that have not reached full maturity and that exhibit high levels of turgor pressure will often exhibit snapping failures (i.e., the stalk will snap in half) during natural lodging events. This failure type is sometimes referred to as “green snap” [3]. In mature maize plants that have stalk lodged this failure pattern involves creasing of the stem near the node line as described in [4]. Stalk lodging causes greater grain losses than root lodging [5]. When stalk lodging occurs before maturity, stalk breakage halts grain filling in the entire plant due to the death of the plant above the breakage site, resulting in yield reduction or even the failure of the entire crop [5–7]. In addition to grain loss, lodging during the dehydration period after physiological maturity (PM) reduces the grain quality and increases harvest costs [8, 9]. Our previous study reported that in mechanical grain harvesting, the maize ear loss increased by 0.15–0.59% for each 1% increase in the lodging rate. Additionally, it was found that the mechanical grain harvesting speed decreased exponentially with increasing lodging rate [10].

The accurate evaluation of the maize lodging resistance in the field can assist in the development of lodging-resistant varieties, the regulation of cultivation measures, and the selection of optimum planting environments. Previous studies on maize stalk lodging focused on aspects of plant morphology, stalk mechanical characteristics, stalk anatomical structure, carbohydrate accumulation and distribution, pests and diseases, planting density, water and fertilizer management, and plant growth regulators [11]. Studies on stalk morphology have shown that maize plants with long basal internodes have a higher ear position and center of gravity than plants with shorter basal internodes, which increases the risk of lodging [12]. In contrast, maize plants with short and thick basal internodes display greater stalk-lodging resistance [13]. About 50 to 80% of the strength of a maize stalk comes from its outer structure, the rind [14]. Several studies have indicated that the rind penetration strength (RPS), crushing strength (CS), and bending strength (three-point bending flexural tests) are all significantly negatively correlated with the stalk lodging rate [15]. Sekhon et al. report that stalk bending strength is strongly associated with maize stalk lodging incidence across multiple environments [16]. Stalk strength is significantly positively correlated with the contents of cellulose, hemicellulose, and lignin [17]. Furthermore, corn borers significantly increase the rate of stalk lodging by drilling into stalks [18], whereas maize stem rot weakens stalk tissue, which greatly increases the risk of stalk lodging [19]. In additionally, the method of mounting specimens almost always has a significant effect on the measured mechanical response [20]. Reliable measurements of stalk bending strength can be obtained by maximizing the span length of bending tests and placing the loading anvil at stronger and denser nodal tissues [21]. There was a different between local and overall compressive moduli of maize stalk [22]. Moreover, as plant density increases, the length of the basal internode significantly increases and the diameter significantly decreases, the contents of cellulose, hemicellulose, and lignin, and the stalk mechanical strength decrease, and the risk of lodging increases [17]. Reasonable water and fertilizer management and the application of plant growth regulators can reduce the internode elongation rate, the ratio of length to diameter, the plant height, and the ear height, promote structural carbohydrate accumulation, and increase stalk mechanical strength and lodging resistance [23, 24]. However, most of these studies were based on the resistance of the plant itself, and less consideration was given to the impact of the external environment on the plant, such as wind. Wind is the primary environmental factor responsible for crop stalk lodging. Stalk lodging occurs when plants are subjected to wind forces greater than the maximum force that the stalk can withstand before breaking. Therefore, the critical wind speed of lodging, which is the synthesized result of wind, leaf area, ear weight, ear height and mechanical properties of main stem internode etc., is needed to evaluate the lodging resistance of plants under different varieties and cultivation practices.

Mechanical grain harvesting is the developing direction of maize production in China [25]. Unlike traditional manual harvesting and mechanical ear harvesting, the mechanical harvesting of maize grain requires grain moisture contents lower than 25% [26]. In mechanical grain harvesting, maize is generally harvested 2–4 weeks after physiological maturity [27]. During maize grain dehydration via plant standing in the field after PM, the risk of lodging increases due to stalk senescence or stalk rot [28, 29]. Nolte et al. estimated that in Ohio, USA, stalk lodging increases by about 5% per week after 15 October and that the ear loss in bushels per acre is equal to an average of about one-third of the percentage of stalk lodging [30]. Additionally, Allen et al. reported that maize harvested late at 15% grain moisture had a 30% lower yield and a 42% higher lodging rate than maize harvested early at 25% grain moisture [31]. The Chinese national standard for mechanical maize grain harvesting (GB/T-21962-2008) suggests that the lodging rate should be less than 5% for such harvesting. In the past, maize harvesting in China was mainly performed by hand and via mechanical ear harvesting, and therefore research on maize lodging has mostly focused on the growth stage before physiological maturity [5,13,17, 23, 32, 33]. After PM, the decomposition of stalk carbohydrate and the decrease of stalk moisture content causes the stalk mechanical strength to decrease [29]. Additionally, at this stage, the leaves senesce and fall off, thus decreasing the windward area and wind force. However, little is known about the critical wind speed of stalk breaking before and after physiological maturity.

Based on previous studies [34–36], this study developed a new type of measurement device to determine maize lodging resistance. The critical wind speed of stalk breaking, the stalk mechanical strength, and the natural stalk lodging rate were investigated in different maize cultivars in order to identify the parameters as evaluate lodging resistance during the later growth stage of maize. Furthermore, the relationship with the stalk mechanical strength, critical wind speed of stalk breaking, and natural lodging rate in the field were analyzed to clarify the factors affecting the critical wind speed of stalk breaking during the late growth stage of maize. The results will help crop breeders develop lodging-resistant maize cultivars.

## Materials and methods

### Experimental design

Field experiments were conducted at the Xinxiang Experimental Station, Chinese Academy of Agricultural Sciences, China (35° 18′ N, 113° 54′ E) during the 2018 and 2019 maize growing seasons. The altitude of the study site is 78 m. The soil was a clay loam and is classified as a Calcareous Fluvisol according the FAO-UNESCO classification system. The soil at 0–20 cm depth had the following characteristics: 18.9 g kg^−1^ organic matter, 78.5 mg kg^−1^ available nitrogen, 21.4 mg kg^−1^ available phosphorus, 162.0 mg kg^−1^ available potassium, and a pH of 8.8. Precipitation, air temperature, and wind speed were measured automatically by a weather station at the experimental site. The monthly weather conditions during the experiment are shown in Table 1.

**Table 1:** Precipitation and temperature during the 2018 and 2019 maize growing seasons at the Xinxiang Experimental Station

Month	Precipitation (mm)		Average temperature (ºC)		Maximum temperature (ºC)		Minimum temperature (ºC)	
	2018	2019	2018	2019	2018	2019	2018	2019
June	122.9	38.2	27.8	27.8	38.4	39.3	14.7	16.7
July	152.4	8.3	28.9	28.7	40.0	38.6	21.2	18.4
August	3.8	54.3	28.0	26.1	38.1	35.9	19.3	14.6
September	92.5	34.3	21.5	21.6	37.0	34.7	9.6	11.9
October	1.0	40.3	16.1	16.3	28.6	33.7	4.3	4.7
November	2.3	1.2	8.6	10.2	20.4	22.9	− 3.1	− 3.2
December	9.0	6.2	1.4	3.4	16.4	9.5	− 11.2	− 8.5

A total of 10 maize cultivars with a wide range of growth stages and a wide range of lodging resistance were planted in 2018. Based on the results for 2018, four widely planted maize cultivars were planted in 2019 (Table 2). In both 2018 and 2019, the sowing date was 13 June and the planting density was 7.5 × 10^4^ plants ha^−1^. Each plot contained 10 rows, each with a length of 10 m and a row spacing of 60 cm. All cultivars were arranged in randomized complete blocks. Each cultivar was replicated three times. A controlled-release fertilizer was applied at 156 kg N ha^−1^, 72 kg P_2_O_2_ ha^−1^, and 60 kg K_2_O ha^−1^ at sowing. Plants were irrigated according to the precipitation and water requirements of high-yield maize [37]. Irrigation was performed when winds were calm. Pesticides were applied as needed to control insect populations. Weeds were periodically removed by hand.

**Table 2:** Experimental cultivars planted in 2018 and 2019

Year	Number of cultivars	Cultivars
2018	10	Zhengdan 958 (ZD958), Xianyu 335 (XY335), Zhongdan 909 (ZD909), Jingnongke 728 (JNK728), Hetian 1 (HT1), Fengken 139 (FK139), Dika 517 (DK517), Dika 653 (DK653), Yudan 132 (YD132), Zeyu 8911 (ZY8911)
2019	4	ZD958, XY335, ZD909, JNK728

### Sampling and measurements

#### Plant morphology

At PM, the plant height (measured from the ground to the top of the tassel) and ear height (measured from the ground to the ear-bearing node) of each cultivar were measured for 10 randomly selected plants in four central rows from each plot using a ruler.

#### Critical wind speed of stalk breaking

**Fig. 1:**
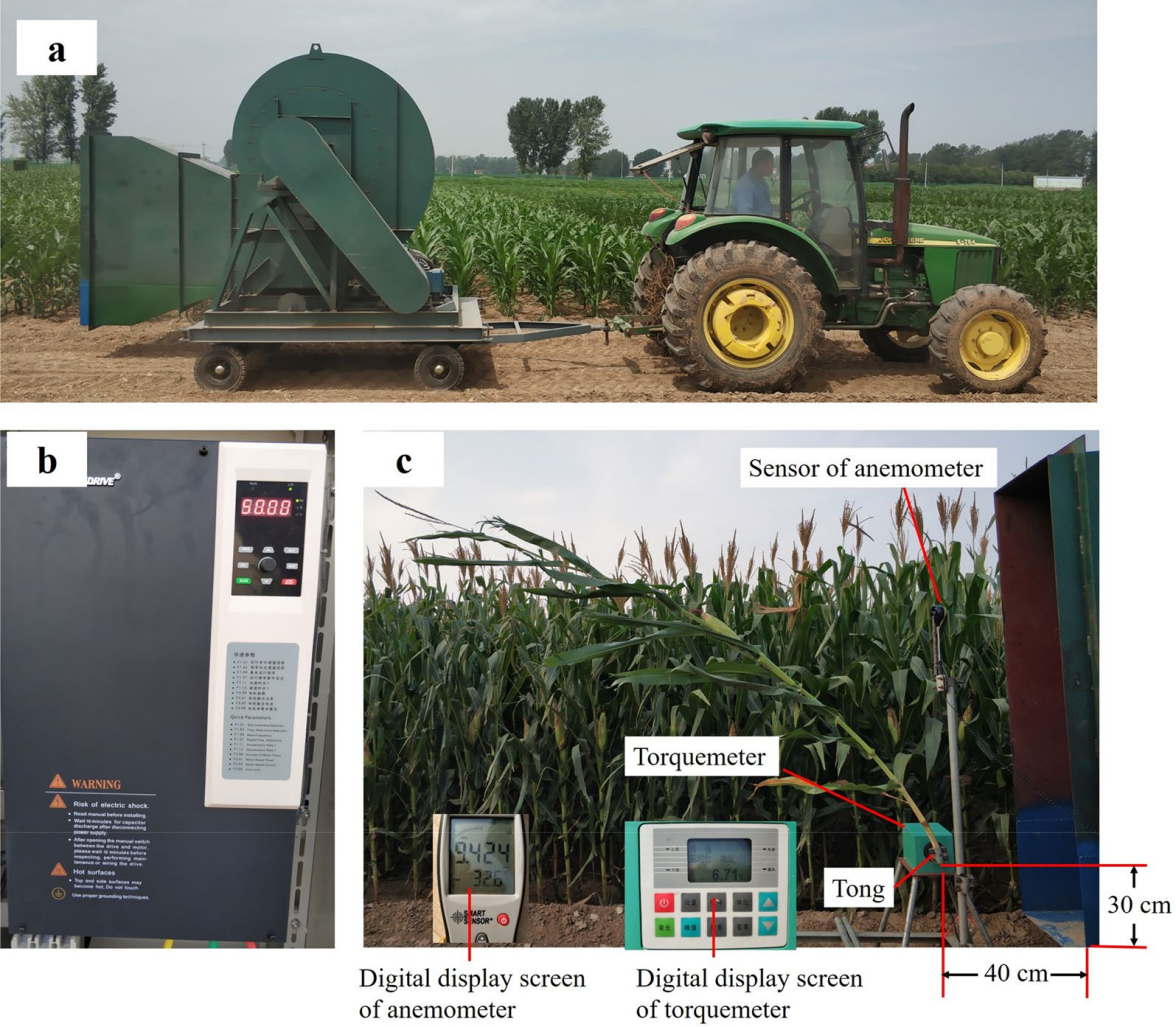
Mobile wind machine used in this study. The system included **a** the supporting structure and electric turbofan, **b** a frequency converter, **c** a plant-fixing structure, digital anemometer, and torquemeter

**Fig. 2:**
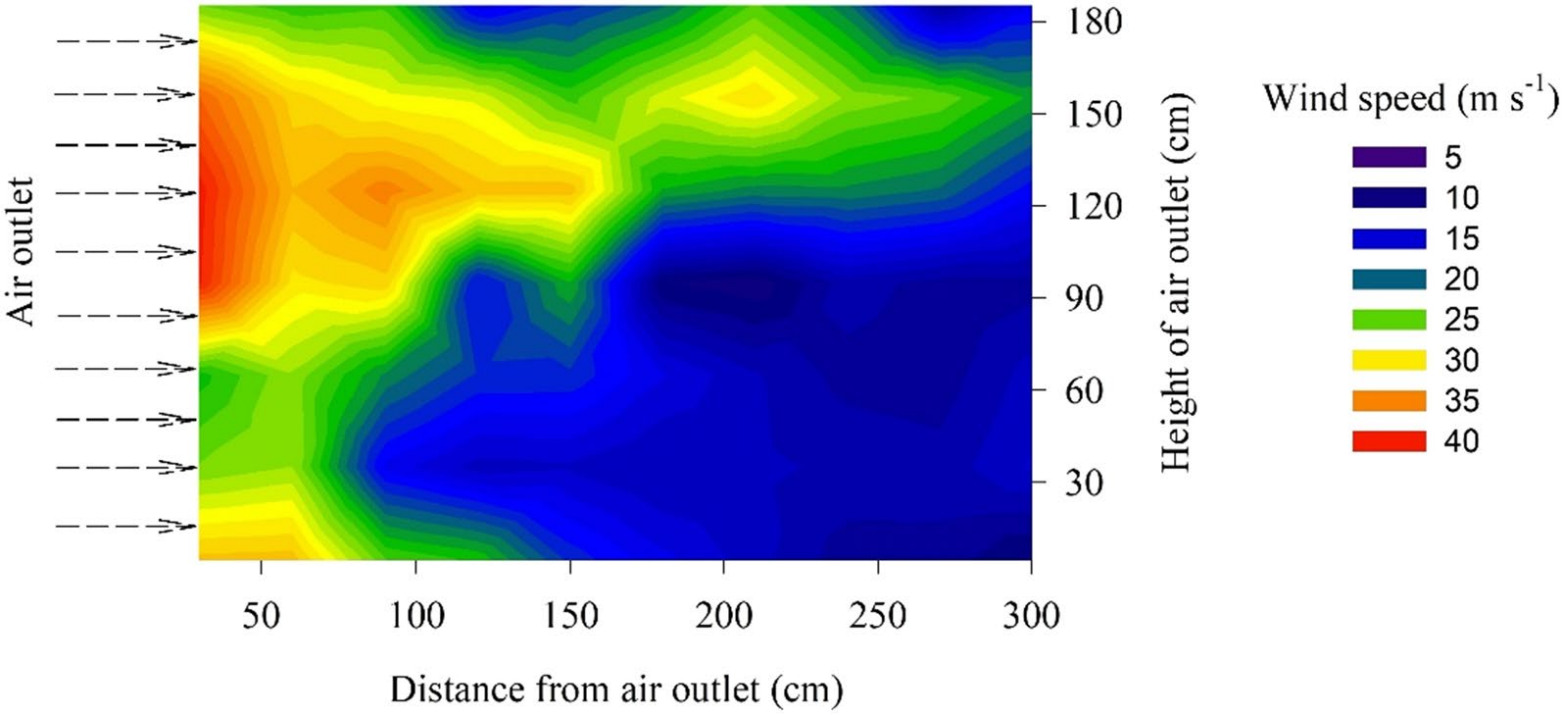
The wind speed (in m s^−1^) from the air outlet in the horizontal and vertical directions

Five maize plants were randomly selected from each plot. The critical wind speed of stalk breaking was determined using a self-constructed mobile wind machine. The mobile wind machine was comprised of a supporting structure, an electric turbofan, a frequency converter, a plant-fixing structure, and a digital anemometer (Fig. 1). The supporting structure was composed of iron plate and four universal wheels, which made the device move in the field. The electric turbofan was fixed with iron plate using the screws. The wind speed of the electric turbofan was controlled by the frequency converter (Fig. 1b). The frequency converter can be set to automatic or manual change. During automatic change, the time from 0 to 50 Hz is 80 s. Meanwhile, for manual change, stepless frequency conversion can be achieved by turning the knob. The plant fixing structure composed of a torquemeter and a tong, which can be used to fix the basal internode of the maize stalk and measure the torque of maize plant as the wind speed increase (Fig. 1c). The input voltage of the inverter motor is 380 V, the power is 55 kW, and the maximum speed is 1100 r min^−1^. Since the maize plant will be bent by the wind, the height of the outlet should be lower than the height of the plant; therefore, we set the height of the outlet to 1.9 m. The wind speeds from the fan outlets in the horizontal and vertical directions under full load was measured. The results show that, in the horizontal direction, the wind speed decreased with increasing distance from the outlet, while in the vertical direction, the wind speed decreased first and then increased with increasing height above the outlet (Fig. 2). At a horizontal distance of 30 cm and a vertical height of 120 cm, the range of controllable wind speed was from 0 to 40 m s^−1^. The coefficient of variation of wind speed among the three repeated measurements was lower than 5%, it was shown that the prototype mobile wind machine is stable and has a controllable wind speed. The total weight of the fan, motor, and supporting structure is about 2.8 tons, which is convenient for transportation.

**Fig. 3:**
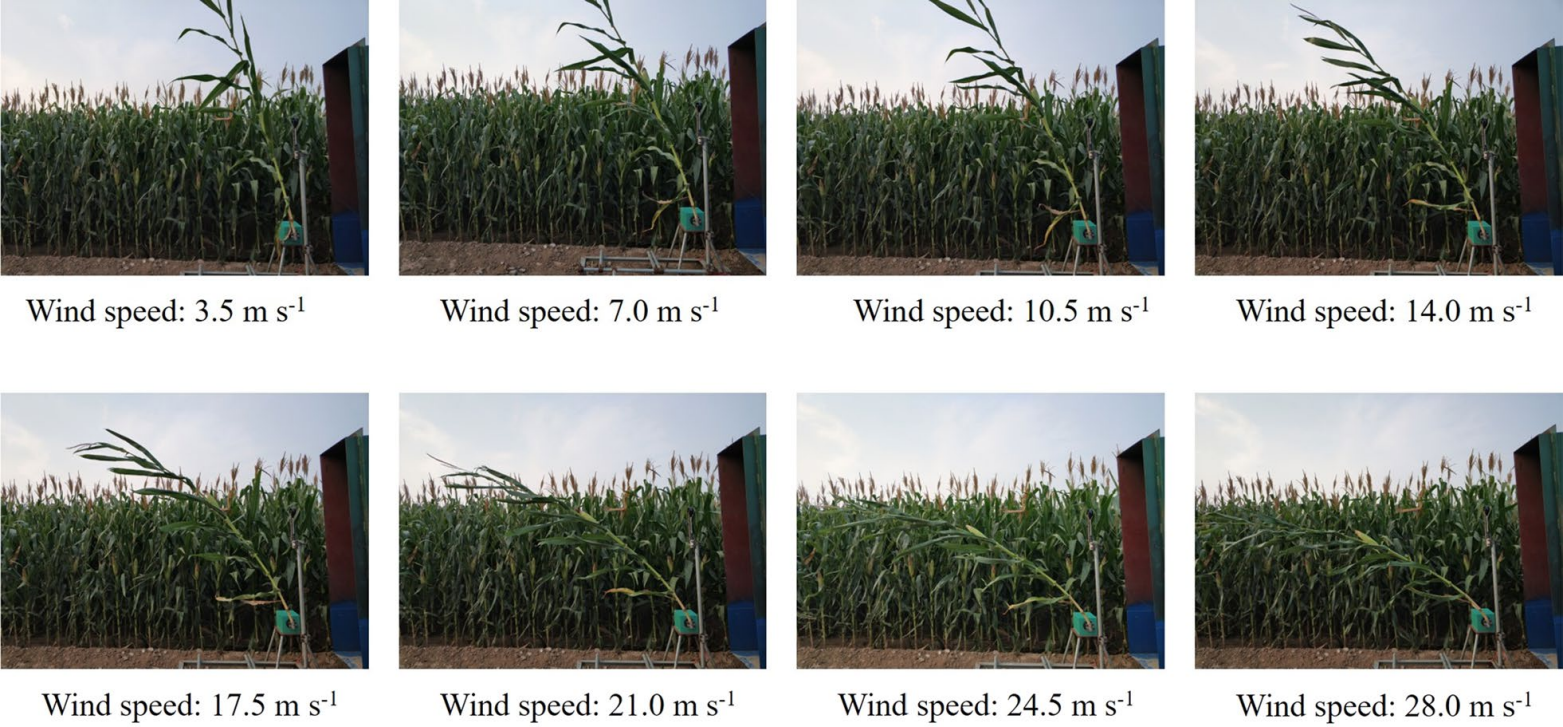
Representation of the measurement of the critical wind speed of stalk breaking in the field

Between physiological maturity of the grain and the time of harvest, naturally lodged corn stalk revealed three failure modes included snapping, splitting, and creasing. About 91% of specimens failed as a result of creasing [38]. Previous studies showed that more than 90% of stalk lodging occurs between the second and fifth elongation internode above the soil [29]. In this study, before measuring the critical wind speed, the maize plant was fixed at the first internode of the stalk above the soil in order to ensure that the plant was oriented vertically under windless condition. During the measurement, the plant was positioned 40 cm away from the air outlet with the bottom of the plant 30 cm above the bottom of the air outlet to ensure the ear within the range of maximum wind speed (Fig. 1c). Stalks that fail in creasing mode typically display either one or two creases, which are oriented perpendicular to the apical-basal axis of the stalk [38]. In this study, the orientation of wind machine was perpendicular to the leaf groove of each stalk. The wind speed was then increased at a uniform rate until the stalk was broken (Fig. 3). The sensor of anemometer was positioned 40 cm away from the air outlet with 120 cm above the bottom of the air outlet. The critical wind speed of stalk breaking was displayed on the screen of the anemometer.

#### Torque of maize plant

Manual change the wind speed, the wind speed was increased in an interval of 3.5–4.0 m s^−1^. Each wind speed level was maintained for a period of 20 s. The value of wind speed and maximum torque were displayed on the screen (Fig. 1c).

#### Stalk rind penetration strength

After measuring the critical wind speed of stalk breaking, the RPS, which is the force required to puncture the stalk rind, was determined with a stalk strength tester (YYD-1, Zhejiang Top Instrument Co., Ltd., Hangzhou, China) according to the method of Xue et al. [39] The stalk strength tester was comprised of a supporting structure, a force gauge with a digital display screen, and a test probe (1 cm in length, 1 mm^2^ cross-sectional area). A stop bar was attached to the test probe so that the probe would only partially penetrate the stalk. Measurements were made in the middle of the internode at its widest side. To collect RPS measurements, the stalk was held firmly and the probe was slowly thrust perpendicularly into the stalk until the stop bar touched the stalk. The highest force exerted during penetration was displayed on the screen and recorded.

#### Stalk breaking force

Five additional maize plants were randomly selected from each plot when measuring the critical wind speed of stalk breaking. For each plant, the breaking force, which is the minimum force required to break the maize stalk, was determined using a stalk strength tester (Zhejiang Top Instrument Co., Ltd.) in the field. To avoid root lodging during the breaking force test, the test was conducted on a sunny day and the soil was compacted beforehand to make sure the plants were firmly anchored in the soil. The direction of the breaking force was always perpendicular to the plant and the position of stalk breaking was recorded [39].

#### Stalk lodging rate

Stalk lodging naturally occurred in the late growth stage. The number of lodged plants was recorded in the middle four rows of each plot along a length of 10 m at the same time as the samples were acquired for the measurement of the critical wind speed of stalk breaking. Plants were considered to be stalk-lodged when they were broken at or below the ear-bearing node [2]. The stalk lodging rate was calculated by dividing the number of lodged plants by the total number of plants in the investigation area.

### Statistical analyses

Statistical analyses were performed using the Predictive Analytics Software (PASW) version 18.0 (IBM SPSS, Somers, NY, USA). Data from each sampling date were analyzed separately. Means were tested using least significant difference tests at the p < 0.05 level (LSD 0.05) in three groups data. Pearson correlations were calculated to identify interrelationships among measured parameters, and path correlation analyses of rind penetration strength (RPS), stalk breaking force, critical wind speed of stalk breaking, and stalk lodging rate were conducted to better understand causal relationships. The stalk lodging rate was affected by maize cultivars being tested, and many cultivars exhibited roughly linear increase in lodging rate over time. Therefore, the data standardization of lodging rate and critical wind speed of stalk breaking was performed before Pearson correlation in order to control for cultivar effects.

## Results

### Growth stage and plant morphology

The difference in the timing of the silking stage of the 10 cultivars was 6 days, the difference in the date of PM was 19 d, and the difference in time from R1 to R6 was 25 days (Table 3). Additionally, plant height and ear height were significantly different among the 10 cultivars. In 2018, cultivar XY335 had the highest plant height and cultivar DK517 had the lowest plant height, while cultivar YD132 had the highest ear height and cultivar FK139 had the lowest ear height. Coefficients of variation (CV) among the 10 maize cultivars were equal to 7% of plant height and 15% of ear height. In 2019, cultivar XY335 had the highest plant height, cultivar ZD958 had the highest ear height, and cultivar ZD909 had the lowest plant height and ear height.

**Table 3:** Plant height and ear height of the studied maize cultivars in different growth stages

Year	Cultivar	Growth stage		Plant morphology	
		Silking	Physiological maturity	Plant height (cm)	Ear height (cm)
2018	FK139	31 July	20 September	244.3 ± 8.5bc	80.3 ± 2.6e
	HT1	30 July	16 September	246.4 ± 2.4bc	99.7 ± 5.9 cd
	JNK728	03 August	23 September	277.5 ± 8.6a	111.4 ± 6.1c
	DK517	02 August	01 October	237.6 ± 7.2c	95.2 ± 8.1d
	YD132	06 August	05 October	282.4 ± 5.3a	136.4 ± 9.4a
	XY335	06 August	07 October	283.3 ± 10.5a	99.0 ± 12.5 cd
	ZY8911	02 August	29 September	255.6 ± 9.5b	97.5 ± 6.4 cd
	DK653	06 August	07 October	273.8 ± 8.6a	125.5 ± 5.7b
	ZD958	05 August	08 October	247.0 ± 6.9bc	107.8 ± 7.8 cd
	ZD909	06 August	09 October	255.0 ± 5.7b	104.6 ± 5.9 cd
2019	ZD958	04 August	01 October	258.0 ± 4.6b	121.8 ± 8.4a
	XY335	06 August	13 October	296.2 ± 9.1a	119.1 ± 5.9a
	JNK728	03 August	11 October	265.8 ± 10.0b	110.4 ± 7.4b
	ZD909	06 August	15 October	246.1 ± 10.8c	99.4 ± 5.1c

Values in the same column followed by different lowercase letters are significantly different at the p < 0.05 level

### Critical wind speed of stalk breaking

**Fig. 4:**
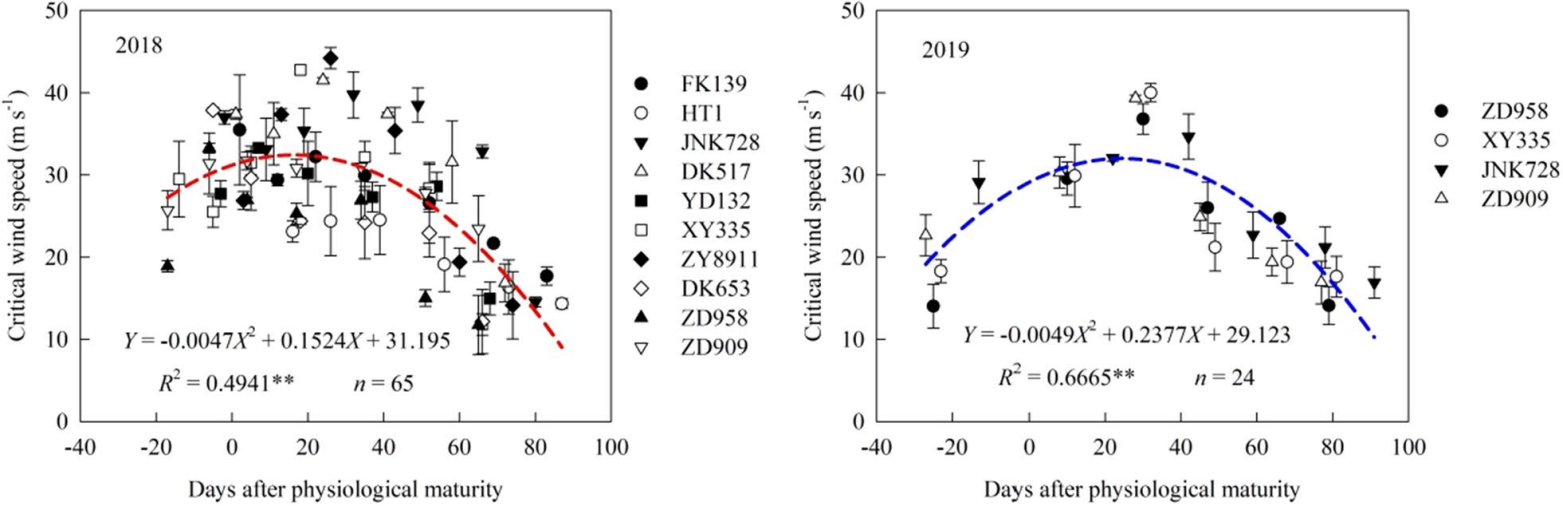
Critical wind speed of stalk breaking before and after physiological maturity for different maize cultivars. **Indicates significance at the p < 0.01 level

The critical wind speed of stalk breaking first increased and then decreased with increasing number of days after physiological maturity (Fig. 4), and was found to follow a quadratic trend. On the quadratic curve which was fitted between the critical wind speed and number of days after PM, the critical wind speed was highest at 16 days after PM in 2018 and at 24 days after PM in 2019. This difference may be due to the fact that the average growth period of the 10 maize cultivars in 2018 was shorter than that of the four cultivars in 2019. In the same measurement period, the critical wind speed of stalk breaking differed among the tested maize cultivars. In 2018, the average critical wind speed of stalk breaking of seven sampling dates was highest for cultivar DK517, followed by cultivar JNK728, while the lowest was for cultivar HT1.

**Fig. 5:**
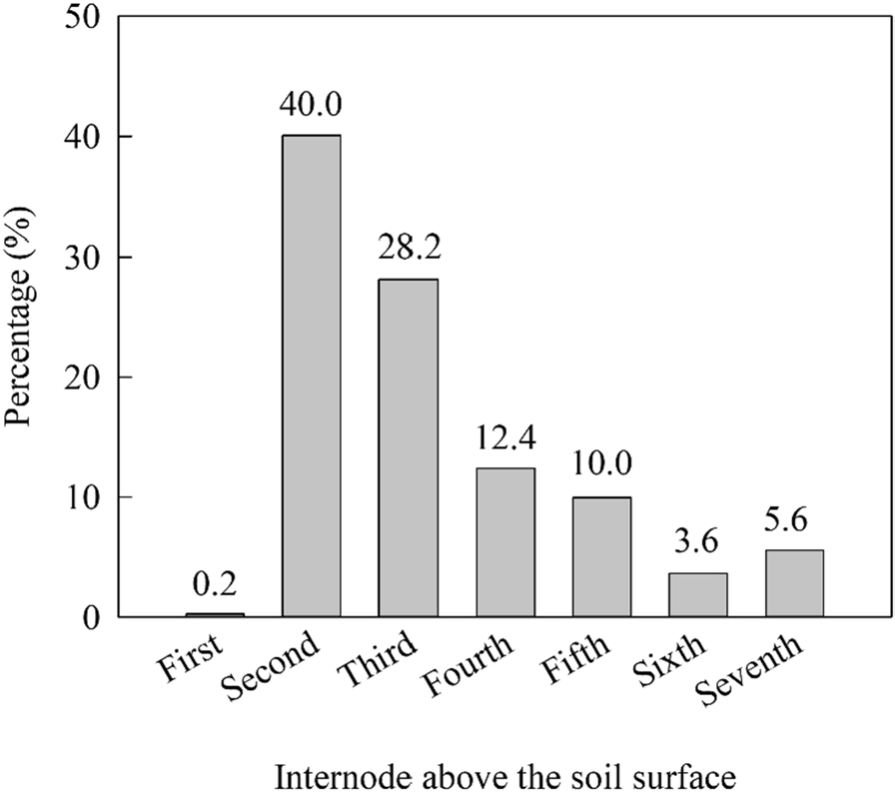
Survey of the position of stalk lodging (n = 421)

The location of failure when using wind machine were collected by 421 plant samples (Fig. 5). The highest proportion of lodging occurred at the second internode, followed by the third internode, and the lowest proportion of lodging occurred at the first internode. Most stalk lodging (90.5%) occurred between the second and fifth internodes.

### Relationship between wind speed and torque

**Fig. 6:**
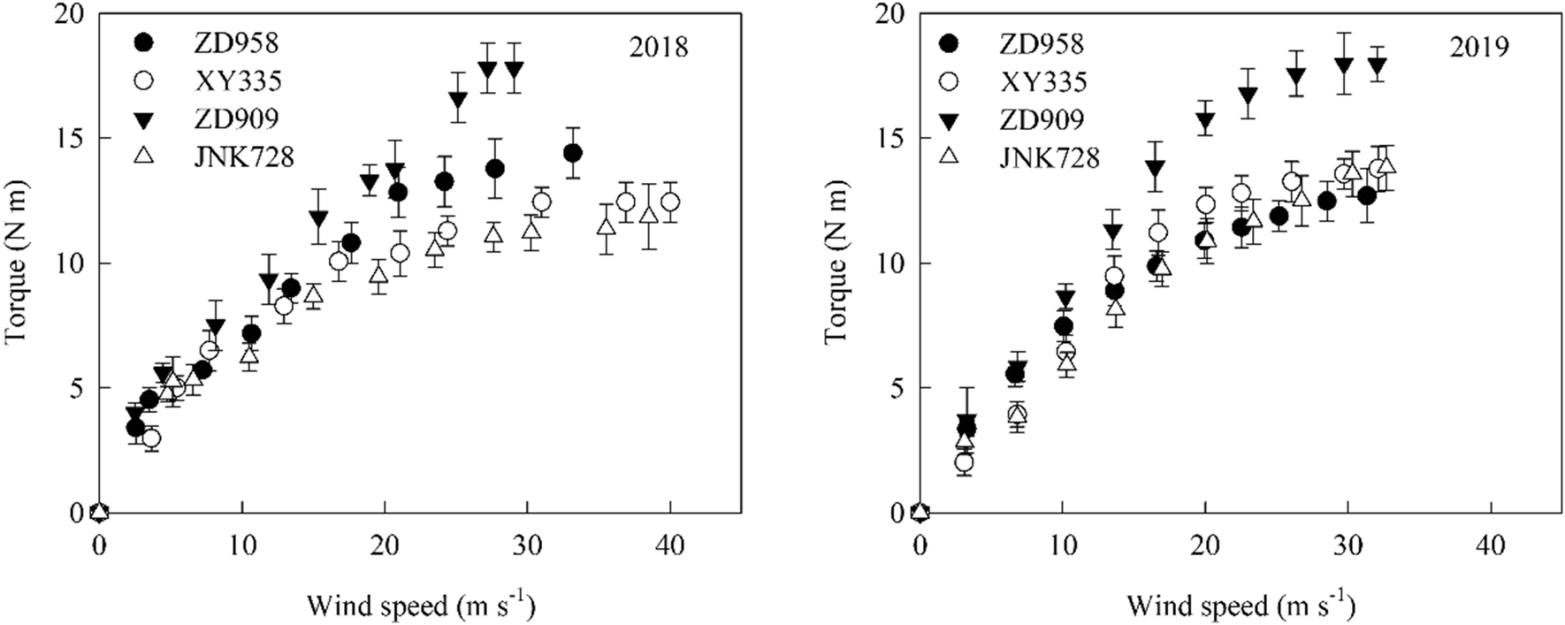
The relationship between torque and wind speed in different maize cultivars (25 October)

The torque at the base of maize plant increased as wind speed increased (Fig. 6). When the wind speed was higher than 15 m s^−1^, the difference of torque between cultivars becomes larger. Under the same wind speed conditions, the cultivar ZD909 had the highest torque. This because of ZD909 had the latest maturity date and highest green leaf area in four maize cultivars when measuring torque at the same date.

### Stalk breaking force

**Fig. 7:**
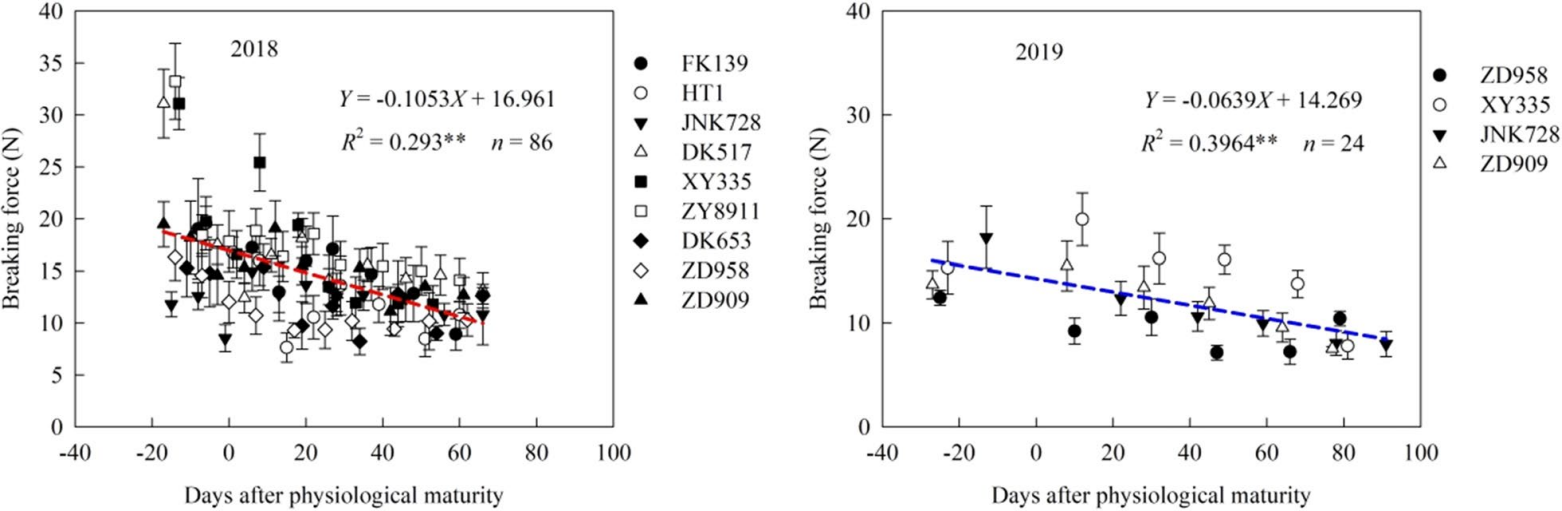
Stalk breaking force before and after physiological maturity in different maize cultivars. **Indicates significance at the p < 0.01 level

The stalk breaking force linearly decreased with increasing number of days after physiological maturity (Fig. 7). In 2018, cultivar ZY8911 had the highest average stalk breaking force across sampling dates and cultivar ZD958 had the lowest. In 2019, the highest and lowest average stalk breaking force was observed in cultivars XY335 and ZD958, respectively.

### Rind penetration strength

**Fig. 8:**
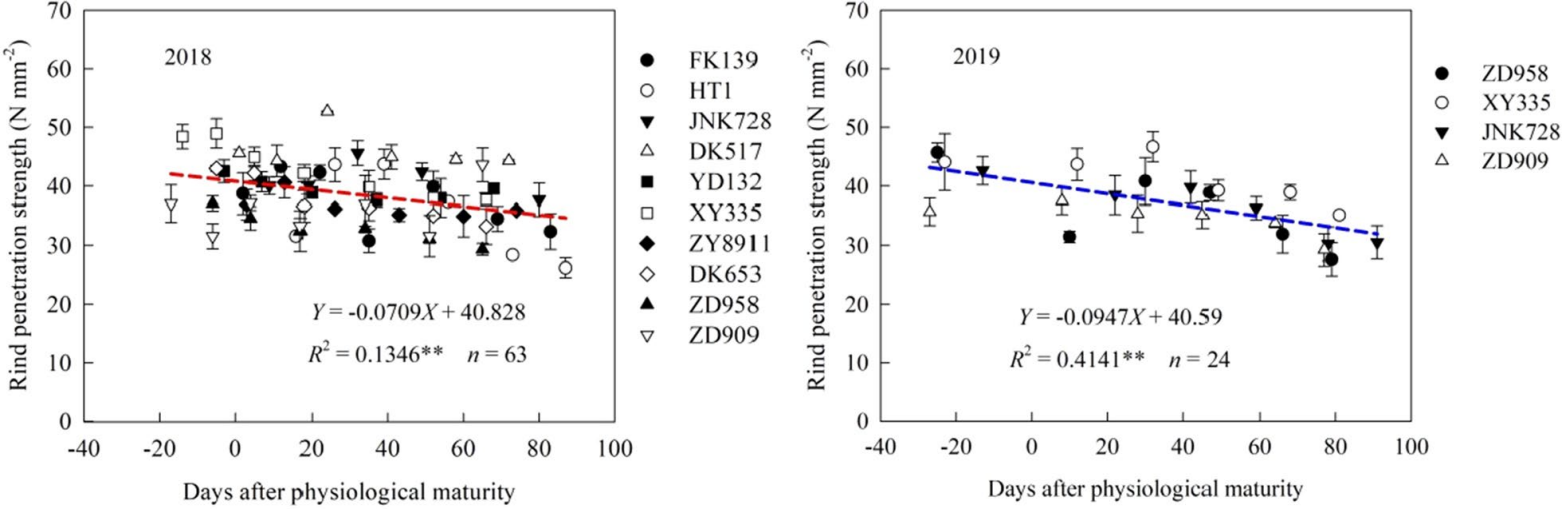
Rind penetration strength (RPS) before and after physiological maturity in different maize cultivars. **Indicates significance at the p < 0.01

The RPS of the third internode above the soil was negatively linearly related with the number of days after physiological maturity (Fig. 8). In 2018, cultivar DK517 had the highest average RPS of the six sampling dates, followed by cultivar XY335, and cultivar ZD958 had the lowest average RPS. In 2019, the average RPS of the six sampling dates was highest in cultivar XY335 and lowest in cultivar ZD909.

**Fig. 9:**
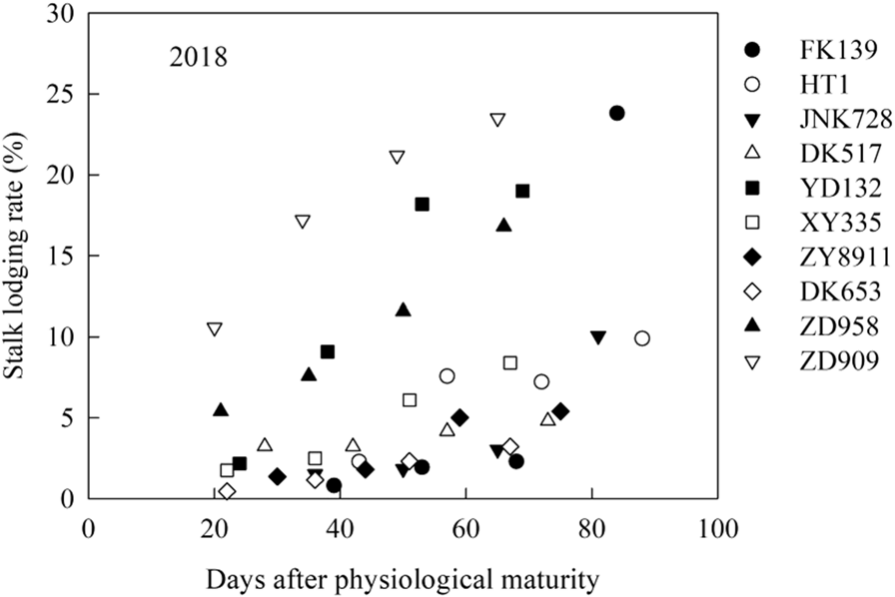
Stalk lodging rate of different maize cultivars in 2018.

### Natural stalk lodging rate

Under natural conditions, the stalk lodging rate in the field gradually increased with increasing number of days after physiological maturity (Fig. 9). The degree of the increase in the stalk lodging rate differed among the studied maize cultivars. In 2018, compared with the first survey (29 October), the largest increase in the stalk lodging rate in the last survey (13 December) was observed for cultivar FK139, and the lowest increase was observed for cultivar DK517. In the last survey, the stalk lodging rate was highest in cultivar FK139, followed by cultivar ZD909, and was lowest in cultivar DK653. There was no lodging naturally occurred in 2019. This because of the wind windy weather in 2019 was lower than that in 2018 during the late stage of maize growth.

### Path analysis

**Fig. 10:**
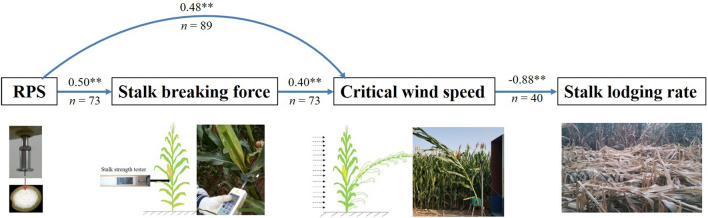
Path analysis of rind penetration strength (RPS), stalk breaking force, critical wind speed of stalk breaking, and stalk lodging rate. ** Indicates significance at the p < 0.01 level.

In 2018, the critical wind speed of stalk breaking was significantly negatively correlated with the stalk lodging rate in the field under natural conditions (Fig. 10). In 2018 and 2019, the critical wind speed of stalk breaking was significantly positively correlated with the stalk breaking force and the RPS. The RPS was significantly positively correlated with the stalk breaking force.

## Discussion

### Critical wind speed of stalk breaking and maize lodging

Crop lodging is determined by two factors: the stress state of the plant and the plant's ability to support its own weight. The plant support system includes the stalk and the roots. It is difficult to accurately assess the lodging resistance of stalks and roots through field observation alone. The use of instruments to perform quantitative measurements in the field allows the direct and objective evaluation of the lodging resistance of plants. Previous studies of the lodging resistance of maize have focused on the plant morphology and stalk strength under different genotypes and cultivation measures. For instance, some researchers have investigated lodging resistance by measuring the plant height, ear height, center-of-gravity height, stalk diameter, and internode length of the basal stalk with a ruler [13, 40] and by measuring the RPS, crushing strength, and bending strength with a stalk strength tester [11,41, 42]. These indicators and methods can reflect the difference in maize lodging resistance for different genotypes and cultivation measures, however, they have certain limitations and one-sidedness. For example, in production, robust plants often have a higher plant height and ear position, which is not conducive to lodging resistance. Nevertheless, robust plants have a higher mechanical stalk strength, which is conducive to lodging resistance. In this study, the critical wind speed of stalk breaking differed greatly between different maize cultivars in the same measurement period. Additionally, there was a significant negative correlation between the critical wind speed of stalk breaking and the natural stalk lodging rate. This suggests that the critical wind speed of stalk breaking is a superior means to determine the stalk loading resistance.

A wind tunnel is the most intuitive and practical means to determine the failure wind speed in order to evaluate crop lodging resistance. Portable wind tunnels were constructed in order to confirm its suitability for the quantitative investigation of the lodging process under various wind conditions and the evaluation of the accuracy of existing theoretical models of plant withdrawal [34, 35]. The company DuPont Pioneer has devised a mobile wind machine that can generate wind speeds of up to 45 m s^−1^ in order to assess stalk lodging under controlled wind conditions and thus facilitate the evaluation of a genotype for maize lodging [36]. Beijing Research Center for Information Technology in Agriculture designs and constructs a mobile wind machine to evaluate the lodging resistance of different cultivars and determine the relationship between lodging resistance and phenotypic traits. However, this mobile wind machine can only move in one direction and therefore the maize must be grown in a specific area [43]. The devices above studies mostly use axial flow fans. This results in winds spread sideways to the nearby open areas under wind machine tests, whereas canopy turbulence is often generated under natural conditions [44]. Additionally, the difference in airflow for different outlet heights of the axial fan is small, which does not match the actual situation in the field, since, under natural conditions, the airflow pressure above the maize canopy is higher than that in the maize canopy [45]. In this study, the device included a turbo fan in order to make the airflow from the air outlet more similar to natural wind. This device has low cost, is convenient to move and simple to operate in the field, and has a high practicality.

### Influencing factors of critical wind speed of stalk breaking

Grain dehydration during plant standing in the field after PM is an important measure to reduce the grain moisture content, broken rate, and impurity rate in mechanical grain harvesting [26]. Additionally, this process reduces grain drying costs and enables planters to obtain greater economic benefits [46]. However, plant standing after PM causes the lodging rate to increase [28]. Previous studies showed that, for 28 maize cultivars grown at 67,500 plants ha^−1^ in Xinxiang, Henan Province, China, a delay in the harvest date from 27 October to 06 December resulted in an increase in the average stalk lodging rate from 0.5% to 11.8% [29]. Further analysis showed that, after maize PM, plant and ear heights no longer change, and the height of the center of gravity decreases due to leaf senescence, resulting in the breakage of internodes and the loss of water in the upper part of the plant. Additionally, leaf abscission decreases the wind force to which the plant is subjected. The changes in morphology described above are meant to improve the stalk resistance after maize physiological maturity.

The RPS reflected the mechanical characteristics of maize stalk, the breaking force comprehensively analyzed the maize ear height and stalk strength. Rind penetrometer resistance is a significant predictor of stalk lodging incidence. However, rind penetrometer resistance lacks sensitivity as compared to stalk bending strength measurements [35, 42]. In this study, the RPS and stalk breaking force decreased with increasing number of days after PM. There was a significant positive correlation between RPS and stalk breaking force. This indicated that, after PM, the degradation of carbohydrates and the decrease of moisture content causes the RPS to decrease, which decreases the stalk breaking force.

This study showed that the critical wind speed of stalk breaking first decreased and then increased with increasing number of days after PM, with the critical wind speed being highest at 16–24 days after physiological maturity. There was a significant negative correlation between critical wind speed and stalk breaking force. Therefore, the change in the stalk-lodging resistance of maize can be divided into two stages after physiological maturity. In the first stage, the leaf senescence rate is quicker than the stalk senescence rate, which reduces the wind resistance of the plants and consequently increases the stalk-lodging resistance. In the second stage, the rapid stalk senescence leads to a rapid decline in the mechanical strength of the maize stalk, which in turn reduces the ability of stalk lodging.

## Limitations

In this study, the position of stalk breaking using new device measuring was highest occurred in second internode above the soil surface. This broken height is lower than that of previous studies, which reported that the highest proportion of stalk lodging was occurred in third internode [29, 47, 48]. This may be related to the fixing position of plant sample in measurement process. The power is 55 kW of the inverter motor. In order to provide sufficient wind speed, we set the heigh of air outlet for the new device is only 1.9 m. This is lower than the plant height of maize in field. In the future, the motor with higher power and speed should be used in mobile wind machine to make sure the height of the outlet is higher than plant height in field. In addition, the testing cycle time was 5 min for one plant sample. In the future, it is necessary to further study to improve the testing efficiency.

The wind is a random load whose speed and direction change over time. Before maize lodging, the plant is caused to vibrate by the influence of wind [49], and when the vibration of a maize plant exceeds a certain limit, the stalk breaks [50]. Maize lodging is affected by wind speed, wind direction, and wind blowing time. This study only studied the critical wind speed of maize stalk breaking under one wind direction. In the future, it is necessary to study the critical wind speed of stalk breaking under different wind directions and different wind blowing times. Maize stalk creases were generally aligned with the major diameter of the stalk cross section and the plants generally fell in the direction of the minor stalk diameter [38]. This may be closely related to plant physiology, the direction of ears and leaves. Therefore, the plant torque and wind speed of stalk breaking among different fixing direction of maize plant should be study in future. This will provide guidance for the plant direction of maize in field.

The lodging rate is extremely problematic because it is often confounded by both long-term and short-term weather events that are different between years. For example, a good growing season with few major wind events may result in low lodging rates while a poor growing season with many major wind events will result in high lodging rates. In this study, the lodging only occurred in 2018, the more date of lodging rate and wind weather should be analysis to determine the critical wind speed in future. In addition, the stress (and therefore bending strength) experienced by the stalk is the result of a dynamic, complex, and interconnected relationship that varies both spatially and temporally between the wind profile, the plant's sail area, the drag coefficient of the plant, and the flexibility of the plant.

## Conclusions

The critical wind speed of stalk breaking can be used to evaluate the stalk-lodging resistance of maize. A new device was comprised of a supporting structure, an electric turbofan, a frequency converter, a plant-fixing structure, and a digital anemometer can be used to measure the critical wind speed of stalk breaking. With increasing number of days after maize physiological maturity, the critical wind speed of stalk breaking increased first and then decreased, reaching a maximum at 16–24 days after physiological maturity. Additionally, in the same measurement period, this critical wind speed differed among the 10 tested maize cultivars. The critical wind speed is affected by leaf area, fresh weight, stalk tenacity, ear height. Further research on wind speed and plant torque, as well as how plant phenotypic indicators affect plant torque.

## Data Availability

The datasets used and/or analyzed during the current study are available from the corresponding author on reasonable request.
